# Neisserial Heparin Binding Antigen (NHBA) Contributes to the Adhesion of *Neisseria meningitidis* to Human Epithelial Cells

**DOI:** 10.1371/journal.pone.0162878

**Published:** 2016-10-25

**Authors:** Irene Vacca, Elena Del Tordello, Gianmarco Gasperini, Alfredo Pezzicoli, Martina Di Fede, Silvia Rossi Paccani, Sara Marchi, Tsisti D. Mubaiwa, Lauren E. Hartley-Tassell, Michael P. Jennings, Kate L. Seib, Vega Masignani, Mariagrazia Pizza, Davide Serruto, Beatrice Aricò, Isabel Delany

**Affiliations:** 1 GSK Vaccines, Siena, Italy; 2 Institute for Glycomics, Griffith University, Gold Coast, Queensland, Australia; University of Kentucky College of Medicine, UNITED STATES

## Abstract

Neisserial Heparin Binding Antigen (NHBA) is a surface-exposed lipoprotein ubiquitously expressed by *Neisseria meningitidis* strains and an antigen of the Bexsero^®^ vaccine. NHBA binds heparin through a conserved Arg-rich region that is the target of two proteases, the meningococcal NalP and human lactoferrin (hLf). In this work, *in vitro* studies showed that recombinant NHBA protein was able to bind epithelial cells and mutations of the Arg-rich tract abrogated this binding. All N-terminal and C-terminal fragments generated by NalP or hLf cleavage, regardless of the presence or absence of the Arg-rich region, did not bind to cells, indicating that a correct positioning of the Arg-rich region within the full length protein is crucial. Moreover, binding was abolished when cells were treated with heparinase III, suggesting that this interaction is mediated by heparan sulfate proteoglycans (HSPGs). *N*. *meningitidis nhba* knockout strains showed a significant reduction in adhesion to epithelial cells with respect to isogenic wild-type strains and adhesion of the wild-type strain was inhibited by anti-NHBA antibodies in a dose-dependent manner. Overall, the results demonstrate that NHBA contributes to meningococcal adhesion to epithelial cells through binding to HSPGs and suggest a possible role of anti-Bexsero^®^ antibodies in the prevention of colonization.

## Introduction

*Neisseria meningitidis* is a strictly human Gram-negative bacterium that is recognized as one of the leading causes of septicemia and bacterial meningitis. It is also an obligate commensal of the nasopharyngeal mucosa, the only known reservoir of infection [1]. Previous studies have revealed that *N*. *meningitidis* is able to adhere to, enter and traffic through epithelial and endothelial cells [1–5]. Adhesion to host epithelial cells and tissues is necessary for bacterial survival, colonization and transmission, and is common to both carriage and disease-causing strains [1]. Disease occurs when an invasive strain crosses the epithelium and enters the bloodstream, causing septicemia, or crosses the blood-brain barrier causing meningitis [1]. *N*. *meningitidis* has evolved numerous surface-exposed adhesive structures that facilitate interactions with human cells. Several adhesins may be present simultaneously and often cooperate to increase the binding avidity necessary for bacterial invasion of host cells [1]. The major adhesive molecules of *N*. *meningitidis* are pili and the outer membrane opacity proteins, Opa and Opc [1, 3]. In addition, other minor surface-exposed proteins have been implicated in adhesion such as NadA [6], Acp [7], NhhA [8], App [9], and MspA [10]. Additional proteins as yet unidentified, however, may also play a role in the interaction of host cells.

Neisserial Heparin Binding Antigen (NHBA or GNA2132) is a surface-exposed lipoprotein that is specific for *Neisseria* species. NHBA is one of the main antigens of the recently developed serogroup B meningococcal vaccine, Bexsero^®^ [11], and is able to induce antigen-specific bactericidal antibodies in both animals and humans [12, 13]. The *nhba* gene is ubiquitous in meningococcal strains of all serogroups and it is also found in *N*. *gonorrhoeae* as well as in different commensal Neisserial species, including *Neisseria lactamica*, *Neisseria polysaccharea*, and *Neisseria flavescens* [14–16]. Analysis of gene sequences from genetically diverse serogroup B strains reveals the existence of more than 400 distinct peptides, for which each is assigned a numerical identifier, which have some association with clonal complexes and sequence types [15, 17]. Considerable variation is observed at the level of the primary amino acid sequence which ranges in length from approximately 430 to 500 residues. In particular, most variability is observed at the level of the amino-terminal region, which is annotated as intrinsically unfolded by commonly used structure prediction algorithms, while the carboxyl-terminal region, represented by a single 8-stranded anti-parallel beta-barrel structure, is highly conserved [18].

The NHBA protein is able to bind to heparin *in vitro* through an Arg-rich region located in a flexible loop between the beta-barrel of the C-terminus and the N-terminus region [12, 18]. This property has been shown to correlate with increased survival of the un-encapsulated bacterium in human serum [12]. Two proteases, the meningococcal NalP and human lactoferrin (hLf), are able to cleave the protein upstream and downstream of the Arg-rich region, respectively, releasing two different fragments [12]: C2, a C-terminal fragment generated by NalP cleavage (as reported in [19]) that contains the Arg-rich domain; or C1, a C-terminal fragment generated by hLf cleavage that lacks the Arg-rich domain. In addition to heparin binding, NHBA has been associated with biofilm formation, whereby the release of the C2 fragment by NalP resulted in a reduction in biofilm formation [20]. Moreover, it was also demonstrated that the C2 fragment alters endothelial cell permeability by inducing the internalization of the adherens junction protein VE-cadherin, which in turn is responsible for leakage of the endothelial barrier that is typically associated with meningococcal sepsis [19].

The aim of the present study was to explore other possible biological roles for NHBA, which might be related to the ability of the protein to bind heparin or heparin-like molecules present on the cell surface and in the extracellular matrix. In particular, we investigated its potential contribution to meningococcal adhesion to epithelial cells, since heparan sulfate binding has often been related to the ability of pathogens to adhere to and invade host cells [21]. Using *in vitro* cellular models, we demonstrated that both the purified recombinant protein and meningococcal strains expressing NHBA are able to interact with epithelial cells by binding to heparan sulfate proteoglycans (HSPGs) through the Arg-rich region of NHBA, showing a new role of NHBA during meningococcal infection. Moreover, anti-NHBA antibodies reduce meningococcal adhesion, suggesting the possibility to impair bacterial colonization by inducing antibodies targeting NHBA through vaccination.

## Materials and Methods

### Bacterial strains and growth conditions

*N*. *meningitidis* serogroup B strains used in this study are listed in Table 1. All *N*. *meningitidis* strains were grown on Gonococcal Medium Base (GC) (Difco) agar plates or in GC broth at 37°C in 5% CO_2_. When required, erythromycin, kanamycin and chloramphenicol were added to achieve a final concentration of 5 μg/ml, 100 μg/ml and 5 μg/ml, respectively. *Escherichia coli* strains DH5α and BL21-DE3 (Invitrogen) were cultured at 37°C in Luria–Bertani (LB) agar or LB broth at 37°C and, when required, ampicillin, kanamycin, and erythromycin were added at final concentrations of 100 μg/mL, 25 μg/mL, and 100 μg/mL, respectively.

**Table 1:** *Neisseria meningitidis* strains used in adhesion assays.

*Strain Isolate*	*Clonal complex*	*ST*	*Year*	*Serogroup*	*Serotype*	*Sero-subtype*	*NHBA peptide*
**MC58**	ST-32 complex/ET-5 complex	74	1985	B	15	P1.7,16b	3
**UK013**	ST-269 complex	275	2001	B	NT	P1.22,9	17
**NGH38**	No value	36	1988	B	NT	P1.3	2
**8047**	ST-11 complex/ET-37 complex	11	1978	B	2	P1.2	20
**M10713**	ST-41/44 complex/Lineage 3	136	2003	B	No value	P1.17,16–3	10

### Constructions of *N*. *meningitidis* knockout and complemented strains

The plasmids pBS-UDgna2132erm or pBS-UDgna2132kan [12] were linearized with ApaI and transformed into *N*. *meningitidis* to generate the *nhba* isogenic knockout mutants (Δ*nhba* strains). The plasmid pBS-c2132cmr was used for *in locus* complementation of the *nhba* gene. Briefly, an upstream flanking region (UP), including the whole *nhba* gene, and a downstream flanking region (DW) were amplified from strain MC58 using the oligonucleotides: 2132UP-FOR/2132UP-REV and 2132DW-FOR/2132DWREV, respectively. The amplified UP fragment was digested with SacI/XbaI, and DW fragment with PstI/KpnI and cloned into pBluescript (Stratagene). The resulting plasmid was digested with XbaI/PstI to insert the chloramphenicol resistance gene, amplified using primers Cm-FOR/Cm-REV. The plasmid DNA to be used for *N*. *meningitidis* transformation was linearized using SpeI. Primers and plasmids used in this study are listed in S1 Table. For transformation by naturally competent *N*. *meningitidis*, ten to fifteen single colonies of a freshly grown overnight culture were suspended in 100 μl of phosphate-buffered saline (PBS) and spotted onto GC agar plates, to which 5–10 μg of linearized plasmid DNA was added, allowed to dry, and incubated for 6–8 hours at 37°C. Transformants were then selected on plates containing erythromycin (5 μg/ml), kanamycin (100 μg/ml), or chloramphenicol (5 μg/ml), and single colonies were grown on selective media for further analysis. Two microliters of crude cell lysates was used as a template for PCR analysis in order to evaluate the correct double homologous recombination event resulting in the knockout of the gene (primers 2132KO-FOR/2132KO-REV) or in the insertion of the complementation cassette (primers 2132SEQ-FOR/2132SEQ-REV).

### Cell fractionation and protein analysis

To prepare total cell extract, *N*. *meningitidis* strains were grown overnight on GC agar plates at 37°C in 5% CO_2_. Colonies from each strain were collected and used to inoculate GC broth to an initial optical density at 600nm (OD600) of 0.05–0.06. The culture was incubated at 37°C with shaking until an OD600 0.5 was reached, and then centrifuged for 5 minutes at 8000 × *g*. The supernatant was discarded and the pellet was resuspended in SDS-containing sample loading buffer. Total lysates were boiled for 5 minutes, separated by SDS-PAGE using the NuPAGE Gel System (Invitrogen) and transferred onto nitrocellulose membranes for Western blot analysis. Western blots were performed according to standard procedures. The different NHBA peptides were identified with polyclonal mouse antisera raised against the recombinant NHBA protein, corresponding to MC58 protein (peptide 3) (diluted 1:1000). Anti-PilE mouse polyclonal serum was used to determine the strain piliation status and a monoclonal anti-Opc antibody (B306) was used to evaluate Opc expression. In all cases, an anti-mouse antiserum conjugated to horseradish peroxidase (Dako) was used as the secondary antibody. Bands were visualized with Super Signal West Pico Chemiluminescent Substrate (Pierce) following the manufacturer’s instructions.

### Cloning and purification of recombinant NHBA proteins

Purification of recombinant NHBA, NHBA ΔRR, NHBA mRR and of recombinant fragments NHBA N1, NHBA N2, NHBA C1 and NHBA C2 has been previously reported [12]. Of note, NHBA N2 and NHBA C2 recombinant proteins correspond to N-his and C-his, respectively, in Serruto *et al* [12], while the same nomenclature was retained for NHBA N1 and C1.

### Cell culture

Hec-1B (human endometrial cells, from *ATCC*^®^ HTB113^™^) were maintained in Modified Eagle Medium (MEM, Life Technologies) supplemented with 150 mM L-glutamine, non-essential amino acids, sodium-pyruvate, antibiotics and 10% heat-inactivated fetal bovine serum (FBS; Gibco). CHO-K1 (*ATCC*^®^ CCL-61) and CHO pgsA-745 (*ATCC*^®^ CRL-2245), which are defective for GAG biosynthesis were maintained in 45% Dulbecco’s Modified Eagle Medium (DMEM, Life Technologies) and 45% F-12 medium (Life Technologies) supplemented with antibiotics and 10% heat-inactivated FBS (Gibco). Calu-3 (human bronchial epithelial cells from *ATCC*^®^ HTB55^™^) were grown in DMEM:F12 (Invitrogen) supplemented with Glutamax (Invitrogen) and 10% FBS. Cells were grown at 37°C with 5% CO_2_.

### Flow cytometry analysis

The ability of anti-capsule mouse polyclonal to bind to the surface of live meningococci was determined using flow cytometry analysis. Bacteria were grown in liquid GC medium to an OD600 of 0.5 and incubated with the anti-capsule mouse polyclonal serum diluted in PBS + 1% BSA (1:3200). After 1 hour, bacteria were washed with PBS + 1% BSA, incubated with a secondary Alexa Fluor 488 goat-anti-mouse antibody (1:1000, Molecular Probes) and fixed with 2% PFA. Cells were analyzed with a Canto II flow cytometer (Beckton-Dickinson) using FlowJo software.

### Immunofluorescence confocal microscopy and immunofluorescence binding assay

For immunofluorescence confocal microscopy analysis, Hec-1B (4 x 10^4^/well), CHO cells or CHO pgsA-745 (2 x 10^4^/well) epithelial cells were seeded on 8-well chamber slides coated with collagen I (BD BioCoat) and cultured for two days. Cells were then incubated with 2 μM of recombinant NHBA (diluted in MEM + 1% FBS) for 90 minutes at 37°C. After washing with PBS to remove unbound protein, cells were fixed with 3.7% formaldehyde (Sigma). Samples were washed three times with PBS + 1% BSA and incubated for 30 minutes with blocking solution (PBS + 3% BSA). Samples were then either stained with anti-heparan sulfate antibody (H1890, US biological) or with anti-chondroitin sulfate monoclonal antibody (CS-56 clone, Sigma), together with an anti-NHBA polyclonal rabbit serum for 1 h at room temperature. After two washes, samples were incubated for 30 minutes at room temperature with the following secondary fluorescent antibodies: Alexa Fluor 568 goat anti-mouse IgG (1:500), Alexa Fluor 488 anti-rabbit (1:500) and Alexa Fluor 647-conjugated Phalloidin (1:200) (Molecular Probes). For staining of NHBA only, anti-NHBA polyclonal mouse serum was used as primary antibody and after two washes, Alexa Fluor 488 goat anti-mouse IgG (1:500) was used as secondary antibody in combination with Alexa Fluor 568 Phalloidin (1:500, Molecular Probes). Glass coverslips were mounted with ProLong^®^ Gold antifade reagent with DAPI (Life Technologies) and analysed using a Zeiss LSM710 confocal microscope. For detection of the internalized C2 purified protein, cells were permeabilised using 0.25% Triton (for 10 minutes at room temperature) before staining with primary anti-NHBA antibody.

To measure binding of recombinant proteins to epithelial cells, cells were seeded on 96-well plates (Corning CellBIND) and cultured for two days. Cells were then incubated with 12, 8, 6, 4, 2, 1, 0.5, 0.26, or 0.013 μM of purified recombinant full length NHBA protein or with 6 μM of the different protein fragments or mutants (diluted in MEM + 1% FBS) for 90 minutes at 37°C. After washing with PBS + 1% BSA to remove unbound protein, cells were fixed with 3.7% formaldehyde (Sigma). Samples were washed three times with PBS + 1% BSA and incubated for 30 minutes with blocking solution (PBS + 3% BSA). Samples were then stained with anti-NHBA polyclonal rabbit sera (1:100) for 1 hour at room temperature. After two washes, samples were incubated for 30 minutes at room temperature with Alexa Fluor 488 anti-rabbit IgG (1:500, Molecular Probes). Fluorescence was measured at 485/535 nm (excitation/emission wavelength) using a Tecan fluorescent plate reader.

### Heparinase and chondroitinase treatment

Hec-1B cells were seeded on 8-well chamber slides (4 x 10^4^/well) coated with collagen I (BD BioCoat) or on 96-well plates and cultured for two days. Cells were then incubated either with 4 U/ml, heparinase III (Sigma) or with 0.25 U/ml chondroitinase ABC (Sigma) dissolved in MEM + 1% FBS at 37°C for 90 minutes and washed once in PBS, before proceeding with binding of the recombinant protein. Efficient removal of heparan sulfate or chondroitin sulfate was checked using mouse monoclonal anti-heparan sulfate antibody (H1890, US biological) or mouse monoclonal anti-chondroitin sulfate antibody (CS-56 clone) and Alexa Fluor 568 goat anti-mouse IgG secondary antibody (1:500), respectively. For quantitative analyses, fluorescence after immunostaining was measured at 485/535 nm (excitation/emission wavelength) using a Tecan fluorescent plate reader.

### Heparin inhibition

Hec-1B cells were seeded on 96-well plates and cultured for two days. Prior to the binding experiment, recombinant full-length NHBA protein (4 μM) was pre-incubated for 1 hour at 37°C with 1 μg/ml, 0.1 μg/ml or 0.01 μg/ml of Heparin (Sigma) diluted in MEM + 1% FBS.

### Adhesion assay

Hec-1B cells (1.5 x 10^4^/well) were seeded in a 24-well tissue culture plate (NUNC) and cultured for two days. The day before the experiment, cells were washed with PBS and incubated with cell medium without antibiotics. Before the infection, cells were washed with MEM containing 1% (vol/vol) FBS (infection medium) and infected with a bacterial suspension at a multiplicity of infection (MOI) of approximately 100. After 3 hours of incubation (at 37°C with 5% CO_2_), the monolayers were washed gently 4 times with infection medium and incubated for 15 minutes at 37°C with 1% saponin (Sigma-Aldrich, United Kingdom). To check the number of viable bacteria recovered, after incubation with saponin, bacteria were collected and plated in GC agar plates in serial dilutions to count colony forming units (CFUs). Statistical analyses were performed using an unpaired T test with Welch’s correction.

Calu3 cells (5 × 10^5^ cm^−2^) were seeded onto collagen coated polyethylene terephthalate cell culture inserts (Transwell, 1 μm pore size, 12 mm diameter) (BD Bioscience) and were allowed to grow and differentiate for 5–6 days. The medium was refreshed every second day. Monolayers reaching transepithelial electrical resistance (TEER) values around 1200 Ω per well, determined using an EVOM TEER meter (World Precision Instruments), were considered fully polarized and were further used for the infection assays. The day before the infection, the medium was replaced with antibiotic-free DMEM:F12 supplemented with Glutamax (Invitrogen) and 1% FBS. Bacteria cultivated overnight on solid media (GC) were harvested and resuspended in antibiotic-free DMEM:F12 and Glutamax without the addition of FBS to reach the desired MOI of approximately 40. After 3 hours of infection (at 37°C with 5% CO_2_), the monolayers were rinsed with infection medium and then treated for 15 minutes at 37°C with 1% saponin. To check the number of viable bacteria recovered after incubation with saponin, bacteria were collected and plated onto solid Mueller Hinton media in serial dilutions to count CFUs. Statistical analyses were performed using an unpaired T test with Welch’s correction.

To test the inhibition of binding by anti-NHBA sera, 10^7^ bacterial CFU were pre-incubated with different dilutions of anti-NHBA mouse polyclonal sera for 1h at 37°C in an Eppendorf tube with gentle shaking. Polyclonal sera obtained by immunizing mice with single recombinant NHBA peptide 3 or NHBA peptide 2 as a fusion protein with NMB1030, which is part of the Bexsero^®^ vaccine [22], were used in the experiments and the pre-immune serum was used as a control of unreactive serum. Bacteria were then washed once with PBS, resuspended in infection medium and added to the cells in the adhesion experiment as described above. Statistical analyses were performed using a one-way ANOVA with Tukey’s multiple comparison test.

### Glycan array analysis

Glycan array slides used in this project were prepared and printed as previously described [23]. Briefly, 1–2 μg of recombinant His-tagged protein was pre-incubated for 10 minutes on ice with mouse anti-His, goat anti-mouse AF555 and rabbit anti-goat AF555 antibodies at a molar ratio of 4:2:1. The array slide was pre-blocked in 0.5% BSA in PBS for 5 minutes, rinsed in PBS and dried before the pre-complexed protein was added for 30 minutes, protected from light. The slide was then washed and dried by centrifugation. Image data was collected using a ProScan Array Microarray scanner (Perkin Elmer) 555ex/568em. The image was analysed using ScanArray Express software (Perkin Elmer) and statistics performed using a Students T-test. Binding was defined as positive if the average fluorescence intensity of the glycan spots was greater than one fold above the adjusted background (average of the slide background plus three standard deviations) in three independent replicates (p<0.001).

## Results

### NHBA binds to epithelial cells through the Arg-rich region

**Fig 1:**
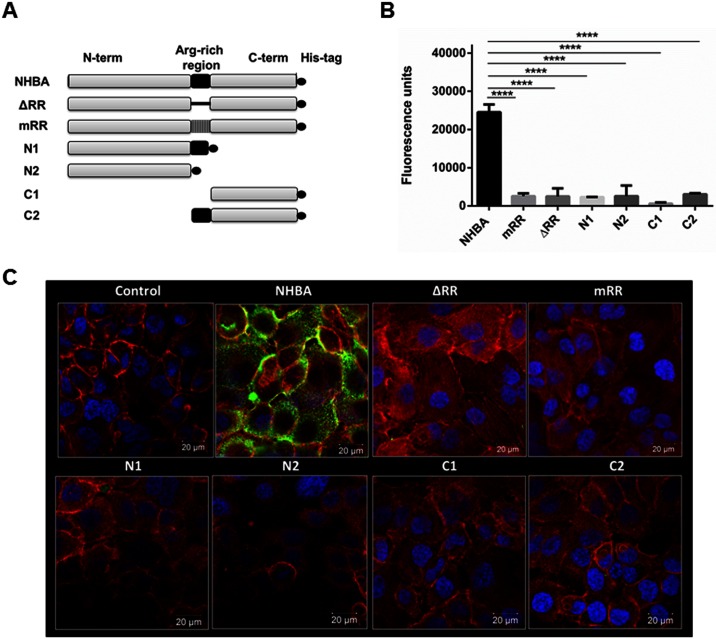
NHBA binds to Hec-1B epithelial cells through its Arg-rich region and NHBA protein fragments are impaired in their ability to bind to Hec-1B epithelial cells. A) Schematic representation of the NHBA recombinant proteins used for the binding assay. The Arg-rich region is represented by a black-box in the central region of the protein, while the black circle after the C-terminus represents the His-tag. In the ΔRR mutant protein, the Arg-rich region has been completely deleted and is represented by a black bar, while in the mRR mutant protein, where the arginine residues contained within the Arg-rich tract have been substituted by glycines, that region is represented by a box with vertical black dashes. N1 and N2 fragments are truncated before or after the Arg-rich region, respectively, while the C1 and C2 start either immediately downstream or upstream of the Arg-rich region. B) Fluorescence of Hec-1B cells after incubation with 6 μM of NHBA, ΔRR or mRR mutants and of the different protein fragments (N1, N2, C1, C2) and immunostaining with a primary mouse polyclonal anti-NHBA serum and secondary fluorescent antibody, using a Tecan reader. Mean fluorescence intensity (MFI) units are reported on the vertical axis. Background fluorescence of cells stained only with primary and secondary antibody was subtracted from all samples. The graph shows the results of one representative experiment performed in triplicate. Each bar represents the mean number of fluorescence units measured, while error bars indicate the standard deviation (n = 3). C) Immunofluorescence confocal microscopy analysis of binding of the wild-type NHBA recombinant protein and the ΔRR or mRR mutants and different protein fragments (N1, N2, C1, C2) to Hec-1B epithelial cells. NHBA proteins were detected with a primary mouse polyclonal anti-NHBA serum and a secondary fluorescent antibody (green staining). As a control, cells were stained with primary and secondary antibody in the absence of NHBA protein. Actin was stained with Phalloidin-568 dye (red staining) and nuclei with DAPI (blue staining).

To explore the biological significance of NHBA binding to heparin, we studied the interaction of this protein with epithelial cells. Hec-1B human epithelial cells were used in *in vitro* binding assays as they have been demonstrated to be a valuable model for *N*. *meningitidis* adhesion [7, 9, 24, 25]. Purified recombinant full length NHBA protein (Fig 1A) corresponding to NHBA expressed by strain MC58, was incubated with sub-confluent monolayers of Hec-1B human epithelial cells. Cells were then stained with anti-NHBA antibodies and fluorescent secondary antibodies and binding was determined by measuring fluorescence intensity (Fig 1B) and visualized through immunofluorescence confocal microscopy (Fig 1C) revealing that NHBA was able to bind to these cells. NHBA wild-type protein was able to bind to cells at low concentrations (0.5 μM) and in a dose-dependent manner, reaching saturation at 6 μM (S1 Fig).

To understand whether this interaction was mediated through the Arg-rich region, which is responsible for the binding to heparin *in vitro* [12], or through other additional protein domains, the same binding experiments were performed with a panel of different NHBA protein mutants and fragments (Fig 1A). Specifically, the two mutants tested were the ΔRR mutant, in which the Arg-rich region was deleted [12], and the mRR mutant, in which all the Arg residues present in that region were substituted with Gly [12]. Moreover, two N-terminal protein fragments (N1 and N2) and two C-terminal fragments (C1 and C2), corresponding to the fragments generated by hLf (N1 and C1) or NalP cleavage (N2 and C2), were also used for the *in vitro* binding analysis [12].

Purity of all the recombinant proteins used was assessed by SDS-PAGE (S2 Fig). Furthermore, Western blot analysis, performed with the same NHBA polyclonal antibody used for immunofluorescence staining, confirmed the same level of recognition across all the purified recombinant proteins (S2 Fig). As shown by immunofluorescence confocal microscopy analysis (Fig 1C) and quantified by measuring fluorescence intensity after immunostaining (Fig 1B), none of the mutants and of the fragments, either containing (N1 and C2 fragments) or lacking the Arg-rich region (N2 and C1 fragments), was able to bind efficiently to epithelial cells compared to the wild-type full length NHBA protein. This result demonstrated that NHBA binding was indeed mediated by the Arg-rich region but that its presence alone is not sufficient for an efficient interaction with epithelial cells. In addition, as C2 was previously shown to be internalized in endothelial cells [19], in another set of experiments, Hec-1B cells were permeabilized before the immunofluorescence staining to determine if it was possible to detect the C2 fragment within epithelial cells. As reported in the S3 Fig, the C2 fragment was shown to be internalized.

### NHBA binds epithelial cells via direct interaction with heparan sulfate proteoglycans

**Fig 2:**
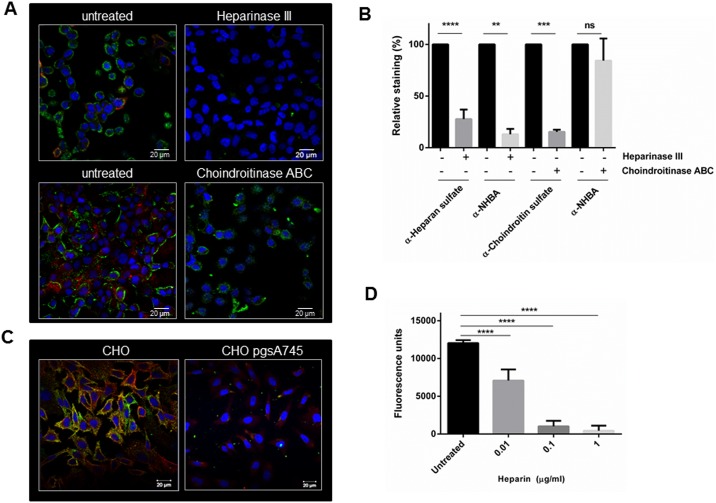
NHBA binds epithelial cells via direct interaction with heparan sulfate proteoglycans. A) Hec-1B epithelial cells were pre-treated with heparinase III or chondroitinase ABC and then used for binding assay with the wild-type NHBA protein. The panels show untreated cells (left) and cells treated (right) with heparinase III (upper panel) or with chondroitinase ABC (lower panel). Cells were stained with a mouse monoclonal anti-heparan sulfate or anti-chondroitin sulfate antibody, respectively, and a secondary fluorescent antibody (red staining). NHBA binding was detected with a primary polyclonal rabbit anti-NHBA serum and a secondary fluorescent antibody (green staining). B) Fluorescence of Hec-1B cells was measured after pre-treatment with heparinase III or choindroitinase ABC and incubation of NHBA, using Tecan fluorescent plate reader. Heparan-sulfate, choindroitin sulfate and NHBA were detected with the same antibodies used for confocal microscopy analysis. Results are reported as percentage of fluorescence with respect to the untreated cells (set to 100%). The graph shows the results of one representative experiment performed in triplicate. Each bar represents the mean number of fluorescence units, error bars indicate the standard deviation (n = 3). NS not significant; ** p value <0.01; *** p value < 0.001; **** p value < 0.0001 (Unpaired t-test with Welch’s correction). C) NHBA protein was untreated or pre-incubated with increasing concentrations of heparin (0.01, 0.1, 1 μg/ml), and then used for binding assays with Hec-1B cells. Cells were then stained with a primary mouse polyclonal anti-NHBA serum and a secondary fluorescent antibody. Mean fluorescence intensity (Fluorescence) units, measured with a Tecan reader, are reported on the vertical axis. The graph shows the results of one representative experiment performed in triplicate. Each bar represents the mean number of fluorescence units, error bars indicate the standard deviation (n = 3). **** p value < 0.0001 (ANOVA test). D) Binding of the wild-type NHBA protein to wild-type CHO-K1 or CHO pgsA-745 epithelial cell monolayers and co-localization with heparan sulfate. NHBA (green staining) and heparan sulfate (red staining) were detected as in panel A. Scale bars represent 20 μm.

Binding of bacterial adhesins to HSPGs present on the cell surface and in the extracellular matrix (ECM) has been related to the ability of pathogens to adhere to and invade host cells [21]. To understand the specific contribution of HSPGs in mediating NHBA binding to epithelial cells, Hec-1B cells were pre-treated with heparinase III, which selectively cleaves heparan sulfate from the cell surface and ECM or with chondroitinase ABC, which removes chondroitin-sulfate residues that are also commonly found in the ECM. After treatment, efficient removal of heparan sulfate or chondroitin-sulfate was confirmed by staining the cells with specific mouse monoclonal anti-heparan sulfate or anti-chondroitin sulfate antibodies and detected by immunofluorescence confocal microscopy (Fig 2A, *red staining*), or by quantification of fluorescence intensity (Fig 2B). Cells were then incubated with the full length NHBA protein and binding was determined by using anti-NHBA antibodies (Fig 2A, *green staining* and Fig 2B). As shown in Fig 2A and 2B, treatment with heparinase III abolished NHBA binding while the interaction was not significantly affected by chondroitinase ABC, indicating that NHBA specifically binds to HPSGs on cell surface.

NHBA binding was also tested using wild-type CHO-K1 or CHO mutant pgsA-745 epithelial cells, which are defective for glycosaminoglycan (GAG) biosynthesis [26]. Immunofluorescence confocal microscopy analysis of CHO cells incubated with the full length NHBA protein revealed that NHBA was able to bind only to wild-type cells, while no binding was observed on CHO mutant cells, devoid of GAG expression (Fig 2C). In addition, as the CHO cells expressed high levels of GAGs, a spatial co-localization of NHBA (labeled in green using anti-NHBA antibody) and heparan sulfate (labeled in red using monoclonal anti-heparan sulfate antibody) was observed, while it was less visible using Hec-1B cells. Pre-incubation of full length NHBA protein with heparin resulted in a significant and dose-dependent reduction of the binding to Hec-1B epithelial cells as compared to the control (Fig 2D), further confirming the specificity of the binding to heparin-like molecules such as HSPGs.

To further analyze the specific binding of NHBA to heparan sulfate, NHBA full length wild-type protein, NHBA mRR and ΔRR mutants, or NHBA fragments containing the Arg-rich region (N1 and C2) were tested for binding on a glycan array containing different HPSG structures (S2 and S3 Tables). Also in this case, the results confirmed that the full length protein, containing an intact Arg-rich region, interacted with heparin, heparan sulfate and their digested fragments. No binding was seen for the fragments and mutants, except for N1 binding to heparan sulfate.

### Deletion of NHBA decreases meningococcal adhesion to epithelial cells

**Fig 3:**
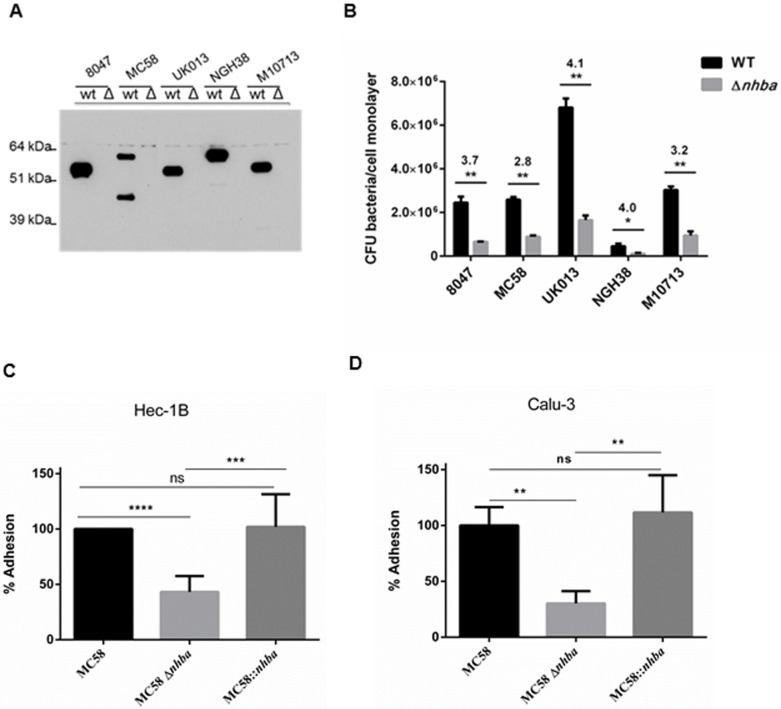
Deletion of NHBA decreases meningococcal adhesion to epithelial cells. A) Western blot analysis of different *N*. *meningitidis* strains expressing NHBA and their corresponding *nhba* isogenic knockout mutants (indicated as WT and Δ, respectively). For strain MC58, the upper band represents the full length NHBA protein and the lower band represents the N2 fragment generated by NalP cleavage. B) Adhesion of a panel of wild-type (WT) and *nhba* knockout mutant (Δ*nhba*) strains to Hec-1B human epithelial cells. The graph reports the average CFUs counted at the end of the assay. Results of one representative experiment performed in triplicate are reported for each strain. Numbers above the bars indicated fold difference in adhesion between WT and Δ*nhba* strains. C and D) Adhesion of the MC58 wild-type strain, *nhba* knockout strain (MC58Δ*nhba*) and *nhba* knockout strain complemented with wild-type *nhba* (MC58::*nhba*) to Hec-1B human epithelial cells (C) or polarized Calu-3 epithelial cells (D). The results are reported as percentage of CFU counts after the end of the assay for each strain with respect to the WT strain (set as 100%). Data represent the means and standard deviations of n = 3 experiments, each performed in triplicate. NS. Not significant. * p value <0.05; ** p value <0.01; *** p value < 0.001; **** p value < 0.0001 (ANOVA test).

To investigate the contribution of NHBA expressed on the meningococcal surface to adhesion to Hec-1B epithelial cells, a panel of *nhba* knockout mutant strains was generated in five different wild-type backgrounds for testing in an *in vitro* adhesion assay. The meningococcal strains were selected in order to cover different NHBA peptides in distinct clonal complexes, all belonging to serogroup B (Table 1). FACS and Western Blot analyses showed that all selected strains were encapsulated and piliated, except for strain 8047 that was encapsulated but non-piliated. Moreover, Western blot analysis showed high levels of Opc expression in MC58 and low levels for strains NGH38 and M10713 (S4 Fig). NHBA expression in the wild-type and knockout strains was also checked by Western blot analysis using total bacterial cell extracts and polyclonal anti-NHBA antibodies (Fig 3A). The results showed that NHBA was expressed in all wildtype strains and highlighted differences in protein length that correlate with amino acid sequence differences previously reported [15]. Moreover, in the case of strain MC58, two bands were detected, one corresponding to the full length protein, and one corresponding to the N-terminal fragment generated by NalP cleavage [12]. The wild-type and isogenic *nhba* knockout mutants were tested in adhesion assays by incubating bacteria with sub-confluent monolayers of Hec-1B epithelial cells. After incubation, adhering bacteria were recovered and quantified by colony forming unit (CFU) counts. As reported in Fig 3B, the wild-type strains tested showed different adhesive capability to epithelial cells, however, in all genetic backgrounds the deletion of *nhba* resulted in a significant reduction of adhesion as compared to the corresponding isogenic wild-type strain, irrespective of differences in NHBA amino acid sequence or NalP cleavage. When the *nhba* gene was complemented *in locus* in MC58 Δ*nhba* mutant strain, the adhesive phenotype was fully restored (Fig 3C). To validate the significance of these findings with respect to meningococcal adhesion to human epithelial cells, the same adhesion assay was performed using Calu-3 polarized human bronchial epithelial cells, a relevant model cell line known to resemble the structure of the polarized epithelium found in the nasopharynx and to express CEACAM receptors involved in adhesion [4, 5]. The results mirrored and confirmed the ones obtained with Hec-1B cells, with the level of adherence of the MC58 Δ*nhba* mutant strain being significantly reduced with respect to the MC58 wild-type and complemented strains (Fig 3D).

### Antibodies against NHBA are able to inhibit meningococcal adhesion

**Fig 4:**
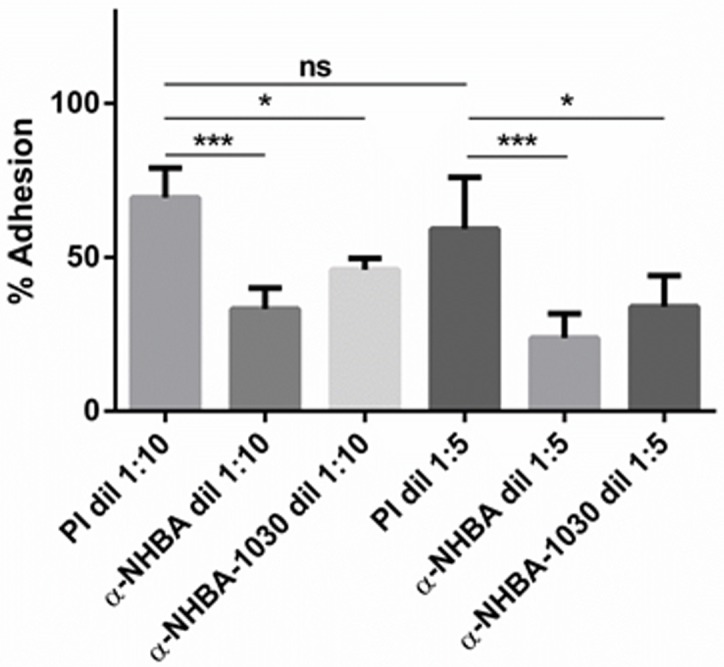
Antibodies against NHBA inhibit meningococcal adhesion. The *N*. *meningitidis* UK013 wild-type strain was pre-incubated with increasing concentrations of a mouse polyclonal serum, raised against the single recombinant NHBA (α-NHBA) or NHBA as a fusion protein with NMB1030, which is part of the Bexsero^®^ vaccine (α-NHBA-1030) and then used in adhesion assays with Hec-1B epithelial cell monolayers. As a control, bacteria were incubated with two dilutions of a pre-immune serum (PI), or without any serum. Results are reported and indicate the percentage of adherent bacteria normalized to the initial inoculum and relative to the control with no serum (set as 100%). Data represent the means and standard deviations of n = 3 experiments, each performed in triplicate. Statistical analyses were performed using a one-way ANOVA with Tukey’s multiple comparison test, comparing each sample pre-incubated with serum to the one pre-incubated with the pre-immune serum, used at the same dilution. NS. Not significant. * p value <0.05; ** p value <0.01; *** p value < 0.001; **** p value < 0.0001.

To further demonstrate the specific role of NHBA in mediating adherence to epithelial cells and to evaluate the possibility to inhibit bacterial adhesion using anti-NHBA antibodies, polyclonal sera obtained by immunizing mice with either the recombinant NHBA (expressed by MC58, peptide 3) or the NHBA component of the Bexsero^®^ vaccine (NHBA peptide 2 expressed as a fusion protein with NMB1030), were used in the adhesion experiment. Bacteria were pre-incubated with the anti-NHBA sera, at different dilutions, for 1 hour before the infection of Hec-1B epithelial cells. The UK013 strain (expressing NHBA peptide 17) was used in this experiment as it showed the highest adhesion to epithelial cells compared to the other wild-type strains tested (Fig 3B). For both anti-NHBA sera tested, the adhesion of UK013 to epithelial cells was effectively reduced in a dose-dependent manner, while no significant inhibition was observed among the negative controls in which bacteria were incubated with different dilutions of pre-immune sera (Fig 4). Of note, similar results were obtained using both sera raised against two different allelic variants of NHBA (peptide 2 or peptide 3 of α-NHBA and α-NHBA-GNA1030, respectively), in the inhibition of adhesion of the UK013 strain that expresses a genetically distant allele of NHBA showing that inhibition of adhesion was not restricted to a specific peptide sequence.

## Discussion

In a previous study, the NHBA protein was shown to bind to heparin *in vitro* through an Arg-rich region and upon binding, un-encapsulated bacteria expressing NHBA showed increased survival in human serum [12]. Heparan sulfate is a close structural homologue of heparin, which is found in the ECM and on the surface of most mammalian cells and is involved in the facilitation of bacterial adhesion to host tissues [21]. In fact, several bacterial adhesins are reported to bind heparin and heparan sulfate, including the *N*. *meningitidis* Opc adhesion [27], *Neisseria gonorrhoeae* Opa proteins [28], heparin-binding hemagglutinin adhesin of *Mycobacterium tuberculosis* [29] and the filamentous hemagglutinin of *Bordetella pertussis* [30].

In this work, we demonstrate that NHBA can bind GAGs (such as heparan sulfate) on epithelial cells thus implicating a new role for NHBA as a meningococcal adhesin. Confocal microscopy analysis showed direct binding of the recombinant NHBA protein to both Hec-1B and CHO epithelial cells. The binding appeared to be mediated specifically by HSPGs, since digestion of Hec-1B cells with heparinase III, but not chondroitinase ABC, abolished NHBA binding to cells and pre-incubation with heparin prevented binding to Hec-1B cells. Furthermore, the NHBA protein did not bind to CHO mutant pgsA-745 cells, which are genetically deficient for GAG biosynthesis, and a co-localization of NHBA with heparan sulfate on wild-type CHO-K1 cells was observed. Interestingly, in polarized epithelial cells HSPGs are mainly expressed on the basolateral surface, therefore it is conceivable to hypothesize that NHBA mediates adherence when these structures become more accessible, i.e once bacteria have transcytosed through the intact mucosal epithelium or after epithelial disruption during inflammation [31]. In accordance with what was previously reported for binding to heparin *in vitro* [12], recombinant NHBA proteins mutated in the Arg-rich region (mRR and ΔRR) were not able to interact with epithelial cells, or heparin, heparan sulfate and their digested fragments printed on glycan arrays, confirming the importance of this region in specifically mediating binding to negatively-charged molecules, such as HSPGs and heparin. The Arg-rich region is conserved among different NHBA peptides, and across all species of *Neisseria* [15], suggesting it plays a key role in the function of NHBA. Importantly, the presence of the Arg-rich region alone was not sufficient for an efficient interaction with epithelial cells. In fact, binding of N1 and C2 fragments, both containing the Arg-rich region, to Hec-1B cells was impaired, suggesting that the Arg-rich tract needs to be presented in the correctly folded full-length NHBA protein in order to allow the interaction with cells and heparin/heparan sulfate structures. Screening of the N1 and C2 fragments for binding to glycan fragments spotted on the glycan array confirmed binding to heparin, heparan sulfate and their digested fragments for the full length protein, while no binding was observed for C2, and N1 bound only to heparan sulfate. In the case of C2, a previous study showed that it rapidly accumulates in the mitochondria of endothelial cell monolayers [19] and we observed its presence within Hec-1B epithelial cells by immunofluorescence confocal microscopy analysis after cell permeabilization. Therefore we cannot exclude that in our binding assays the interaction of C2 fragment on the surface of epithelial cells may not be detected due to rapid endocytosis of this fragment by the cells.

Analyzing the adhesion to Hec1B epithelial cells in a panel of five distinct strains of *N*. *meningitidis* we observed that the adhesion phenotype was independent of the NHBA peptide expressed or the presence or absence of Opc expression or the piliation status of the strain. Moreover, NHBA cleavage by NalP in the MC58 strain was not sufficient to abrogate NHBA-mediated adhesion, perhaps due to the incomplete cleavage shown by Western blot analysis that left sufficient full length protein on the bacterial surface to enable adhesion. A recent study showed that due to phase variation, NalP progresses to a phase-off status during carriage, suggesting that there may be a selective advantage to reducing the activity of NalP during persistent colonization [32, 33]. These data are in agreement with the need to have a full length NHBA on the bacterial surface to enable interaction with HSPGs on epithelial cells during colonization. Interestingly, it has been demonstrated that NHBA contributes to biofilm formation by binding to extracellular DNA an important component of the meningococcal biofilm matrix [20]. DNA-binding is a common property of heparin-binding molecules, as heparin is also characterized by a surface negative charge density. Our new finding that NHBA binds to HPSGs on epithelial cells suggests that NHBA might have a double role in bacterial colonization in the nasopharynx, by directly mediating adhesion of bacteria to epithelial cells on one hand, and by helping the formation of biofilm and meningococcal microcolonies on the other.

Of note, in *N*. *meningitidis* strains belonging to clonal complex ET-5, such as MC58, the sequence upstream of the NHBA coding sequence contains a 150-bp region identified as a Contact Regulatory Element of Neisseria (CREN). This regulatory element, specific for pathogenic *Neisseria* species, is involved in the induction of the downstream associated genes upon contact with target eukaryotic cells and it is necessary for optimal adhesion of meningococcus to epithelial cells [34]. NHBA expression is known to be induced after incubation of bacteria with epithelial cells in the MC58 strain (that contains the CREN region) and not in the 8013 strain (where the CREN region is absent) [35], suggesting that cell contact increases NHBA levels on meningococcal surface in the ET-5 invasive hyper-virulent strains. For future studies, it will be interesting to expand the analysis of *nhba* gene expression and regulation in different host niches and in particular under *in vitro* conditions that mimic the nasopharynx, or *in vivo*.

Overall, the presented data suggest that NHBA has distinct roles in different steps of meningococcal pathogenesis. NHBA has already been reported to influence bacterial survival in blood through binding to heparin, and to contribute to vascular leakage by altering endothelial permeability [12, 19]. Here we showed for the first time that NHBA specifically contributes to host-pathogen interaction via the ability to bind to HSPGs present in the ECM and on the cell surface, thus representing a new addition to the repertoire of meningococcal adhesins.

An important finding in this study is the dose-dependent inhibition of meningococcal adhesion by anti-NHBA polyclonal antibodies. This not only confirms further the specificity of the interaction, but suggests that anti-NHBA antibodies, that could be elicited either from bacterial infection or vaccine-induced responses, may interfere with interaction of meningococci with HSPGs on epithelial cells and hence colonization by NHBA-expressing bacteria. In our experiments, inhibition of adhesion was not restricted to a specific peptide sequence, as anti-NHBA from two specific allelic variants inhibited UK013 expressing a genetically distant variant of NHBA. A recent carriage study among university students in the UK showed that vaccination with Bexsero^®^ reduced meningococcal carriage rates during 12 months after vaccination [36]. Thus, it is tempting to suggest that anti-NHBA antibodies raised against the NHBA antigen that is part of Bexsero^®^ vaccine could be responsible for the reduction of meningococcal colonization and carriage of diverse circulating strains.

## Supporting Information

S1 Table Plasmids and primers used in this study. (DOCX)

S2 Table Glycan array analysis recombinant NHBA protein fragments. (DOCX)

S3 Table Structures of heparin/ heparan sulfate and heparinase digests investigated by glycan array analysis. (DOCX)

S1 Fig Titration of binding of NHBA to Hec-1B cells. Fluorescence of Hec-1B cells after incubation with increasing concentrations of full length NHBA protein and immunostaining with a primary polyclonal anti-NHBA antibody and secondary fluorescent antibody, using a Tecan reader. Arbitrary fluorescence units are reported on the vertical axis. Background fluorescence of cells stained only with primary and secondary antibody was subtracted from all samples. The graph shows the results of one representative experiment performed in triplicate. Each point represents the mean number of fluorescence units measured in the triplicate wells for each concentration tested. Error bars show the standard deviation of three measurements.
(TIF)

S2 Fig Expression of purified recombinant NHBA proteins. A) Coomassie blue staining of full-length NHBA, NHBA mutants ΔRR and mRR, and of NHBA protein fragments N1, N2, C1, C2 loaded on 4–12% polyacrilamide gel. B) Western blot analysis of full-length NHBA, NHBA mutants ΔRR and mRR, and of NHBA protein fragments N1, N2, C1, C2 using a polyclonal mouse anti-NHBA serum.
(TIF)

S3 Fig C2 internalization in Hec-1B epithelial cells. Immunofluorescence confocal microscopy analysis of binding of the C2 purified recombinant protein to Hec-1B epithelial cells. C2 protein was detected with a primary mouse polyclonal anti-NHBA serum and a secondary fluorescent antibody (green staining), after a permeabilization step. As a control, cells were stained with primary and secondary antibody in the absence of protein. Actin was stained with Phalloidin-568 dye (red staining) and nuclei with DAPI (blue staining).
(TIF)

S4 Fig Characterization of the strains used for adhesion assays to epithelial cells. Western Blot analysis of different *N*. *meningitidis* strains expressing NHBA and their corresponding *nhba* isogenic knockout mutants (indicated as WT and Δ, respectively) using a monoclonal anti-Opc antibody (A) or anti-PilE polyclonal serum (B). C) FACS analysis of different *N*. *meningitidis* strains expressing NHBA and their corresponding *nhba* isogenic knockout mutants using an anti-capsule polyclonal serum. RFI, relative fluorescence intensity. Filled grey profiles represent bacteria incubated without primary antibody. Red profiles indicate capsule expression in the WT strain, while green profiles represent that of their *nhba* isogenic knockout mutants.
(TIF)
