# Supplementary material for: Neisserial Heparin Binding Antigen (NHBA) Contributes to the Adhesion of Neisseria meningitidis to Human Epithelial Cells
Source: PLoS One. 2016 Oct 25;11(10):e0162878. doi: 10.1371/journal.pone.0162878 (PMC5079597; doi:10.1371/journal.pone.0162878)
Supplement: S1 Table — (DOCX) [file pone.0162878.s001.docx]

**S1 Table. Plasmids and primers used in this study**

| **Plasmids** | | |
| --- | --- | --- |
| **Name** | **Relevant characteristics** | **Reference or source** |
| pBS-UDgna2132erm | plasmid used to generate the *nhba* isogenic knockout mutants | Serruto et al 2010 |
| pBS-UDgna2132kan | plasmid used to generate the *nhba* isogenic knockout mutants | Serruto et al 2010 |
| pBS-c2132cmr | plasmid used for in locus complementation or the *nhba* gene | This study |
| **Primers** | | |
| **Name** | **Sequence 5’- 3’** | **Restriction sites** |
| 2132UP-FOR | gagaGAGCTCGGGTATCTACTCGCAAAGCGGTTTG | SacI |
| 2132UP-REV | gagaTCTAGATGTTGATGCCGTCTGAAGCGAGGGC | XbaI |
| Cm-FOR | gagaTCTAGAGGATCCGAATTCTACTCTCGACAG | XbaI |
| Cm-REV | gagaCTGCAGGGATCCGTGATATAGATTGAAAAG | PstI |
| 2132DW-FOR | gagaCTGCAGTGCGGACTTGAACCGGACCCGCGA | PstI |
| 2132DW-REV | gagaGGTACCGGCGTTTATGCCTTCTTTACCCG | KpnI |
| 2132KO-FOR | CGCCCTCGCTTCAGACGGCATCAACA | - |
| 2132KO-REV | GGGTCGCGGGTCCGGTTCAAGTC | - |
| 2132SEQ-FOR | GGGTATCTACTCGCAAAGCGGTTTG | - |
| 2132SEQ-REV | GGCGTTTATGCCTTCTTTACCCG | - |
