# Supplementary material for: Neisserial Heparin Binding Antigen (NHBA) Contributes to the Adhesion of Neisseria meningitidis to Human Epithelial Cells
Source: PLoS One. 2016 Oct 25;11(10):e0162878. doi: 10.1371/journal.pone.0162878 (PMC5079597; doi:10.1371/journal.pone.0162878)
Supplement: S2 Table — (DOCX) [file pone.0162878.s002.docx]

**S2 Table.** **Glycan array analysis of recombinant NHBA protein fragments**

| **Structure** | **Glycan information/source** | **NHBA protein** | | | | |
| --- | --- | --- | --- | --- | --- | --- |
|  |  | **FL** | **mRR** | ***∆*RR** | **N1** | **C2** |
| **Heparan Sulfate** | Polymer mix of structures, containing disaccharides of II-S, I-A, IV-A listed below | ✔ |  |  | ✔ |  |
| **Heparin** | (GlcA/IdoAα/β1-4GlcNAcα1-4)n (n=200) | ✔ |  |  |  |  |
| **Heparin disaccharide I-S sodium salt**  **α-ΔUA-2S-[1→4]-GlcNS-6S** | C_12_H_15_NO_19_S_3_Na_4_  (Predominant disaccharide produced from heparinase I and II digestion of heparin) | ✔ |  |  |  |  |
| **Heparin disaccharide II-S sodium salt**  **α-ΔUA-[1→4]-GlcNS-6S** | C_12_H_16_NO_16_S_2_Na_3_  (Produced from heparinase II digestion of heparin and heparin sulphate) |  |  |  |  |  |
| **Heparin disaccharide III-S sodium salt**  **α-ΔUA-2S-[1→4]-GlcNS** | C_12_H_16_NO_16_S_2_Na_3_  (Produced from heparinase I and II digestion of heparin) | ✔ |  |  |  |  |
| **Heparin disaccharide I-A sodium salt**  **α-ΔUA-2S-[1→4]-GlcNAc-6S** | C_14_H_18_NO_17_S_2_Na_3_  (Minor component produced from heparinase II digestion of heparin) | ✔ |  |  |  |  |
| **Heparin disaccharide II-A sodium salt**  **α-ΔUA-[1→4]- GlcNAc-6S** | C_14_H_19_NO_14_SNa_2_  (Produced from heparinase II and III digestion of heparin and heparin sulphate) |  |  |  |  |  |
| **Heparin disaccharide III-A sodium salt**  **α-ΔUA-2S-[1→4]- GlcNAc** | C_14_H_19_NO_14_SNa_2_  (Minor component produced from heparinase II digestion of heparin) | ✔ |  |  |  |  |
| **Heparin disaccharide IV-A sodium salt**  **α-ΔUA-[1→4]-GlcNAc** | C_14_H_20_NO_11_Na  (Produced from heparinase III digestion of heparin sulphate) | ✔ |  |  |  |  |

A tick indicates binding, which is defined as positive if the average fluorescence intensity of the 4 repeat glycan spots is greater than one fold above the adjusted background (average of the slide background plus three standard deviations) in three independent replicates (Students T-test p<0.001). Glycans abbreviations: ΔUA, 4,5 unsaturated uronic acid residue; GlcA, glucuronic acid; IdoA, idouronic acid; GlcNAc, *N*-acetyl glucosamine; GlcNS, glucosamine-*N*-sulfate; 2S, 2-*O*-sulfate; 6S, 6-*O*-sulfate; Na, sodium. NHBA full length wild type protein (FL), Arg-rich region deletion mutant (ΔRR), Arg-rich region mutant (mRR), N1 and C2 fragments containing the Arg-rich region.
