# Supplementary material for: Neisserial Heparin Binding Antigen (NHBA) Contributes to the Adhesion of Neisseria meningitidis to Human Epithelial Cells
Source: PLoS One. 2016 Oct 25;11(10):e0162878. doi: 10.1371/journal.pone.0162878 (PMC5079597; doi:10.1371/journal.pone.0162878)
Supplement: S3 Table — (DOCX) [file pone.0162878.s003.docx]

**S3 Table.** **Structures of heparin/heparan sulfate and heparinase digests investigated by glycan array analysis**

| **Glycan** | **Glycan Structure** |
| --- | --- |
|  |  |
| **Heparan Sulfate** |  |
| **Heparin** |  |
| **Heparin disaccharide I-S sodium salt**  **α-ΔUA-2S-[1→4]-GlcNS-6S** |  |
| **Heparin disaccharide II-S sodium salt**  **α-ΔUA-[1→4]-GlcNS-6S** |  |
| **Heparin disaccharide III-S sodium salt**  **α-ΔUA-2S-[1→4]-GlcNS** | **** |
| **Heparin disaccharide I-A sodium salt**  **α-ΔUA-2S-[1→4]-GlcNAc-6S** |  |
| **Heparin disaccharide II-A sodium salt**  **α-ΔUA-[1→4]- GlcNAc-6S** |  |
| **Heparin disaccharide III-A sodium salt**  **α-ΔUA-2S-[1→4]- GlcNAc** | **** |
| **Heparin disaccharide IV-A sodium salt**  **α-ΔUA-[1→4]-GlcNAc** |  |

Abbreviations: ΔUA: 4,5 unsaturated uronic acid residue, GlcNAc: *N*-acetyl glucosamine, GlcNS: glucosamine-*N*-sulfate, 2S : 2-*O*-sulfate, 6S: 6-*O*-sulfate, Na: sodium
