# Supplementary figures and images for: Neisserial Heparin Binding Antigen (NHBA) Contributes to the Adhesion of Neisseria meningitidis to Human Epithelial Cells
Source: PLoS One. 2016 Oct 25;11(10):e0162878. doi: 10.1371/journal.pone.0162878 (PMC5079597; doi:10.1371/journal.pone.0162878)

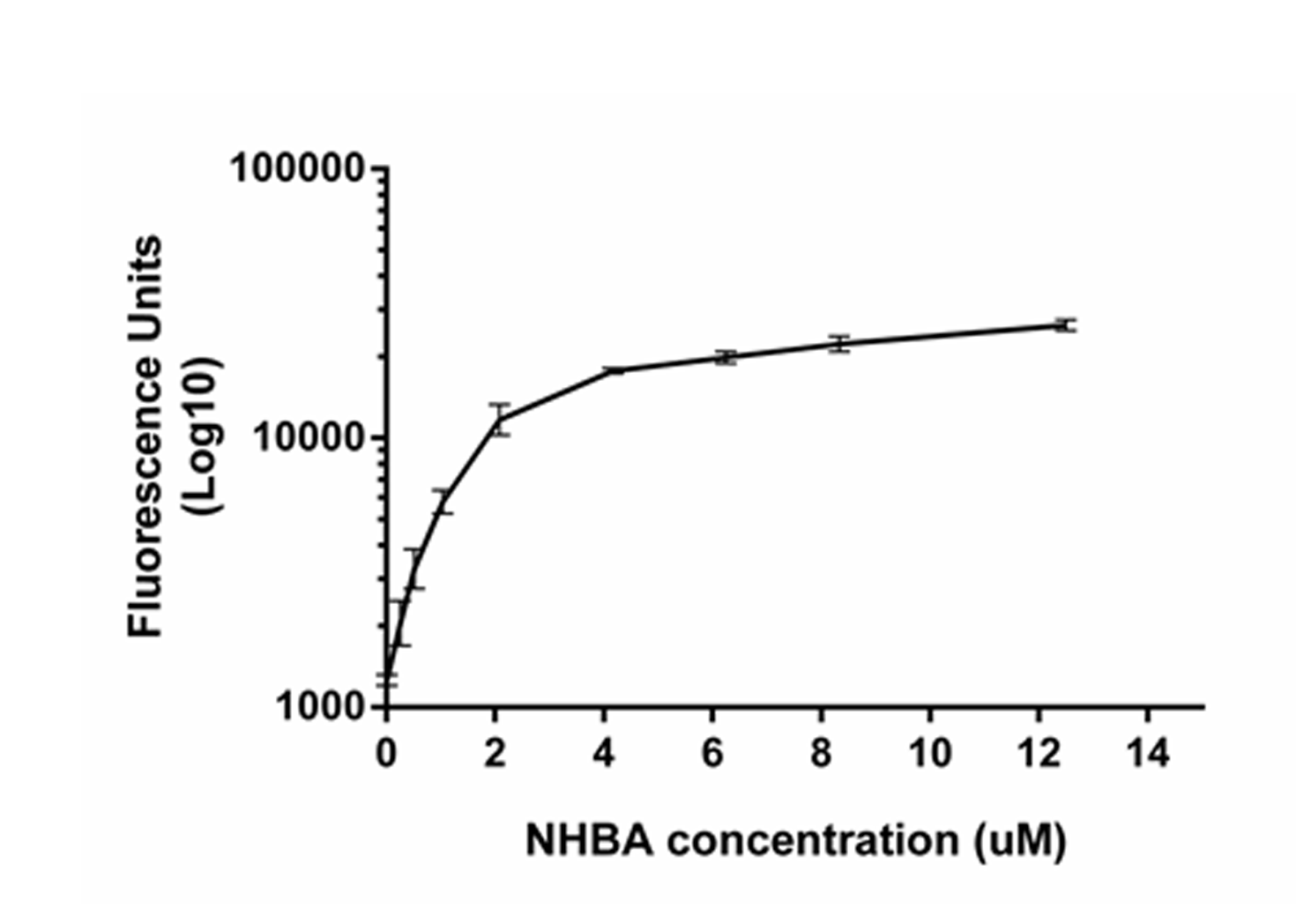

Supplement: S1 Fig — Fluorescence of Hec-1B cells after incubation with increasing concentrations of full length NHBA protein and immunostaining with a primary polyclonal anti-NHBA antibody and secondary fluorescent antibody, using a Tecan reader. Arbitrary fluorescence units are reported on the vertical axis. Background fluorescence of cells stained only with primary and secondary antibody was subtracted from all samples. The graph shows the results of one representative experiment performed in triplicate. Each point represents the mean number of fluorescence units measured in the triplicate wells for each concentration tested. Error bars show the standard deviation of three measurements. (TIF) [file pone.0162878.s004.tif]

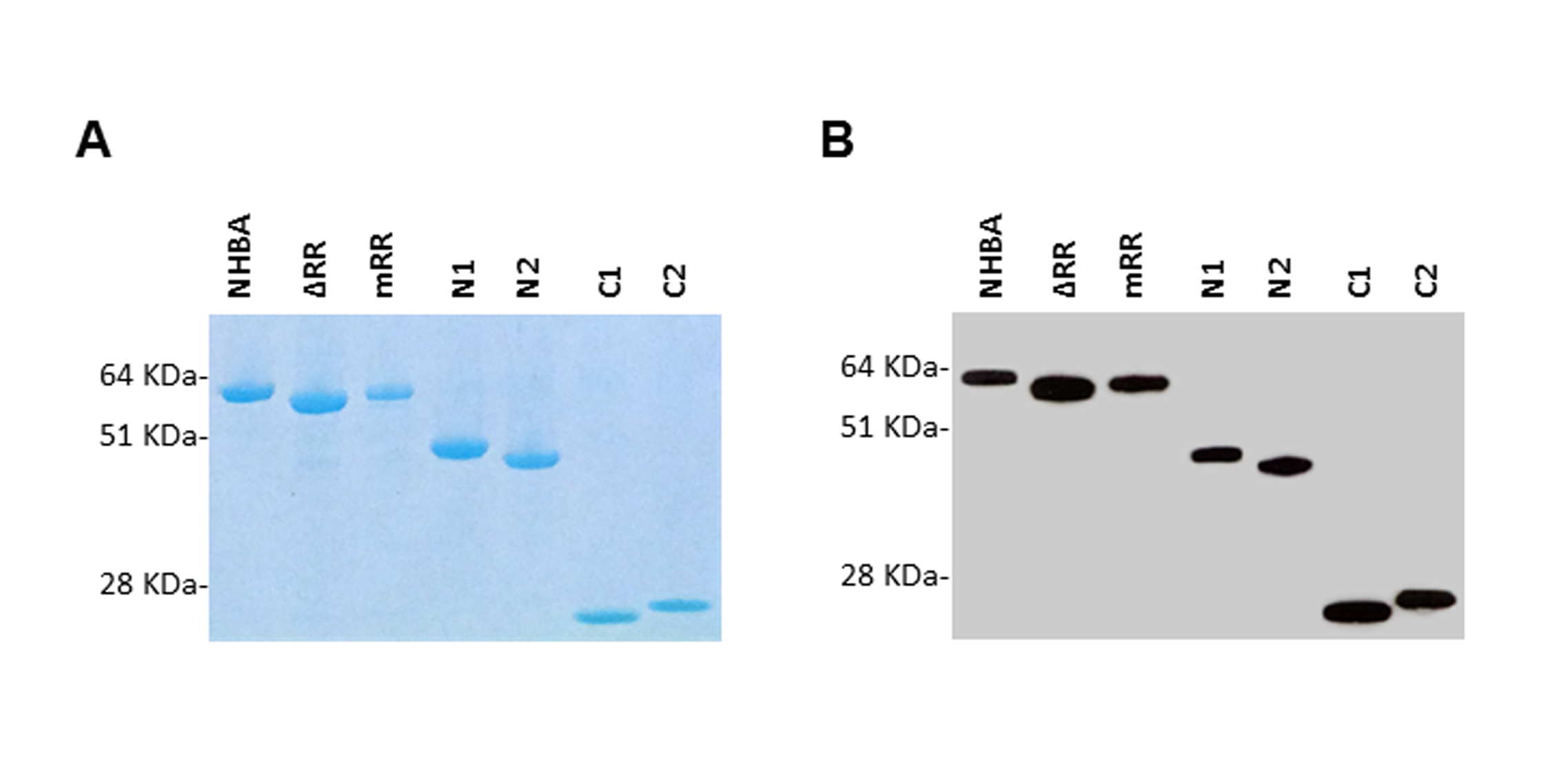

Supplement: S2 Fig — A) Coomassie blue staining of full-length NHBA, NHBA mutants ΔRR and mRR, and of NHBA protein fragments N1, N2, C1, C2 loaded on 4–12% polyacrilamide gel. B) Western blot analysis of full-length NHBA, NHBA mutants ΔRR and mRR, and of NHBA protein fragments N1, N2, C1, C2 using a polyclonal mouse anti-NHBA serum. (TIF) [file pone.0162878.s005.tif]

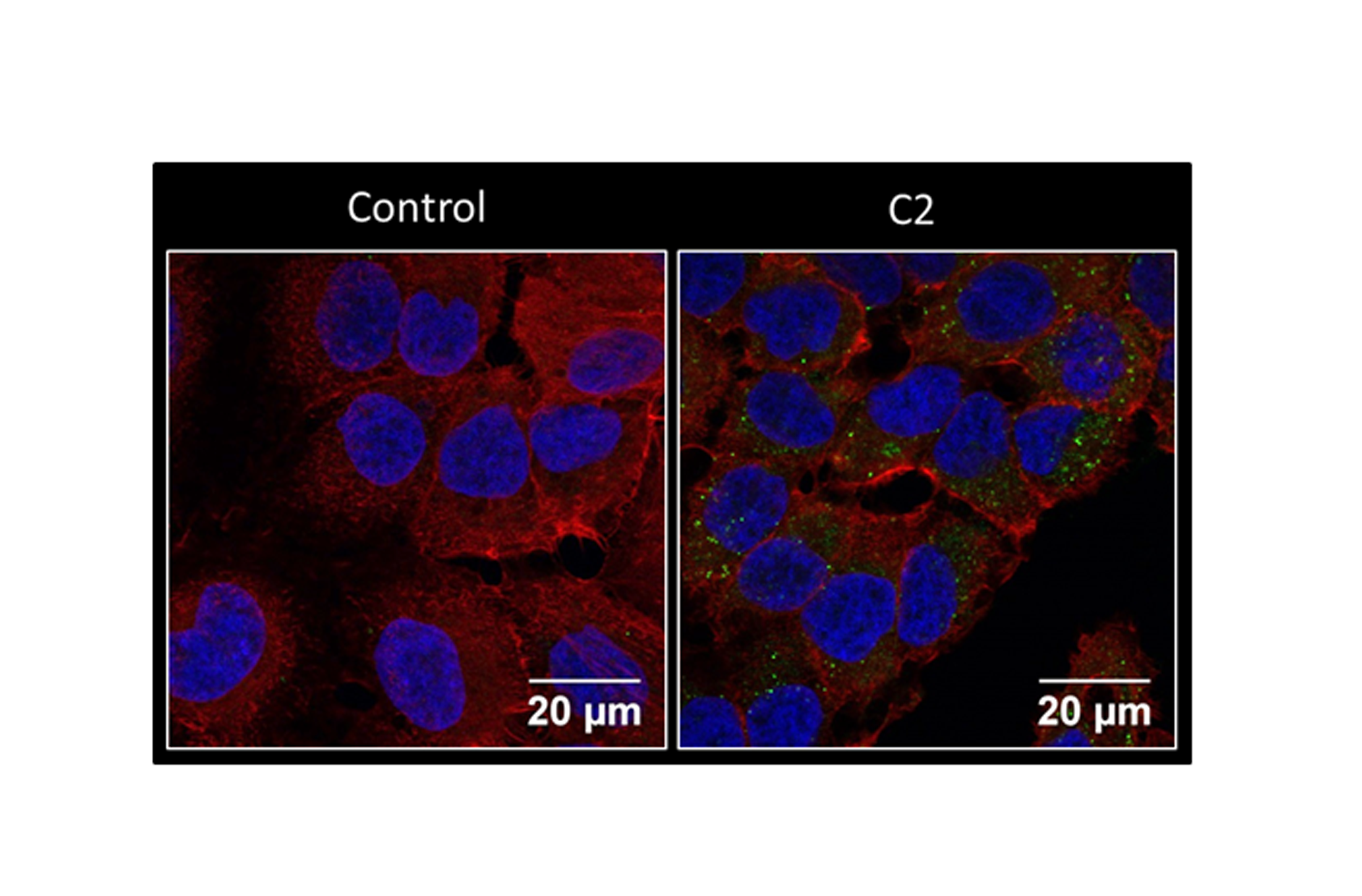

Supplement: S3 Fig — Immunofluorescence confocal microscopy analysis of binding of the C2 purified recombinant protein to Hec-1B epithelial cells. C2 protein was detected with a primary mouse polyclonal anti-NHBA serum and a secondary fluorescent antibody (green staining), after a permeabilization step. As a control, cells were stained with primary and secondary antibody in the absence of protein. Actin was stained with Phalloidin-568 dye (red staining) and nuclei with DAPI (blue staining). (TIF) [file pone.0162878.s006.tif]

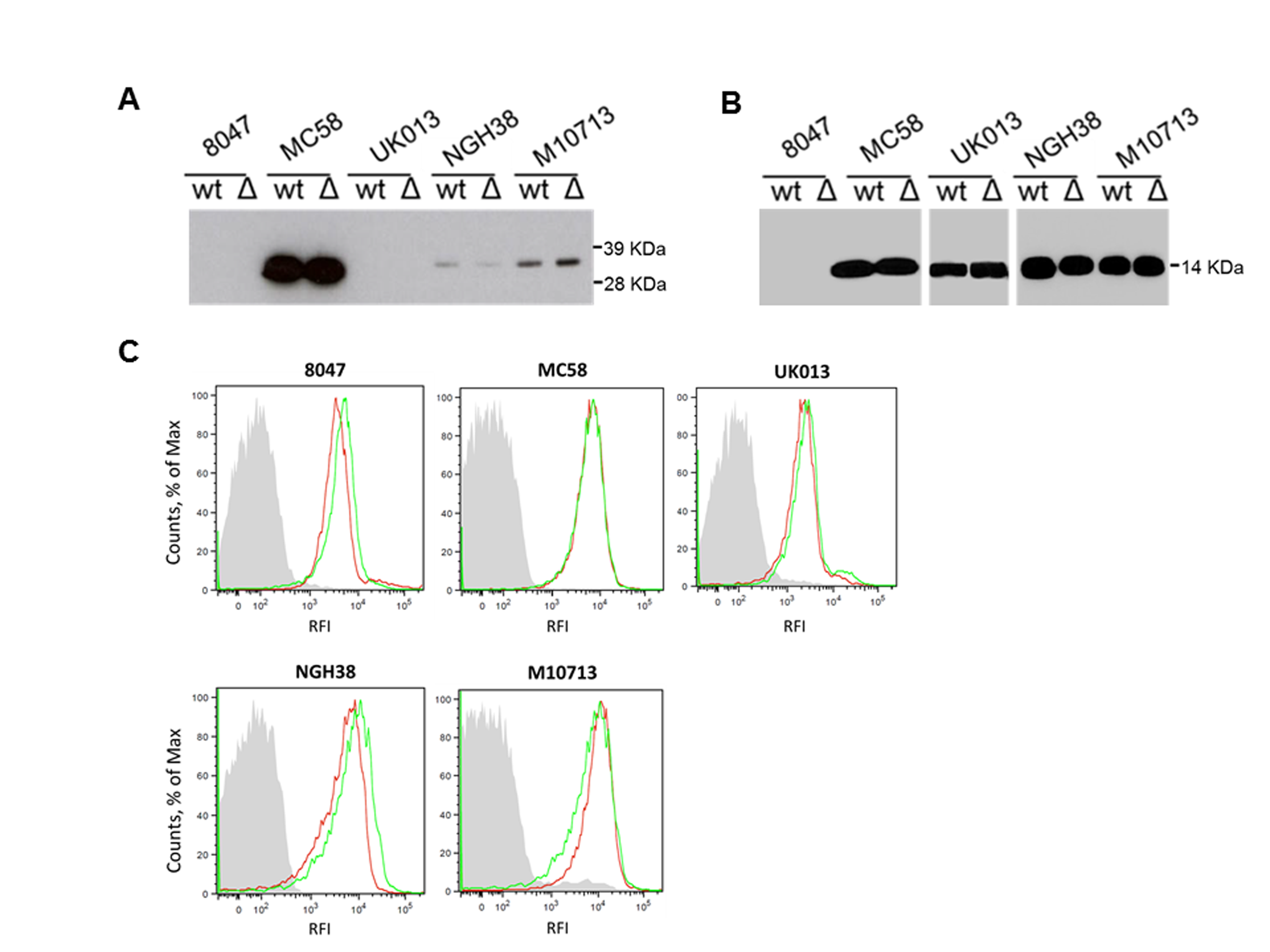

Supplement: S4 Fig — Western Blot analysis of different N. meningitidis strains expressing NHBA and their corresponding nhba isogenic knockout mutants (indicated as WT and Δ, respectively) using a monoclonal anti-Opc antibody (A) or anti-PilE polyclonal serum (B). C) FACS analysis of different N. meningitidis strains expressing NHBA and their corresponding nhba isogenic knockout mutants using an anti-capsule polyclonal serum. RFI, relative fluorescence intensity. Filled grey profiles represent bacteria incubated without primary antibody. Red profiles indicate capsule expression in the WT strain, while green profiles represent that of their nhba isogenic knockout mutants. (TIF) [file pone.0162878.s007.tif]
